# Estimating neonatal length of stay for babies born very preterm

**DOI:** 10.1136/archdischild-2017-314405

**Published:** 2018-03-27

**Authors:** Sarah E Seaton, Lisa Barker, Elizabeth S Draper, Keith R Abrams, Neena Modi, Bradley N Manktelow

**Affiliations:** 1 Department of Health Sciences, University of Leicester, Leicester, UK; 2 Neonatal Unit, University Hospitals of Leicester NHS Trust, Leicester, UK; 3 Neonatal Data Analysis Unit, Section of Neonatal Medicine, Department of Medicine, Imperial College London, London, UK

**Keywords:** neonatal, neonatal intensive care, length of stay

## Abstract

**Objective:**

To predict length of stay in neonatal care for all admissions of very preterm singleton babies.

**Setting:**

All neonatal units in England.

**Patients:**

Singleton babies born at 24–31 weeks gestational age from 2011 to 2014. Data were extracted from the National Neonatal Research Database.

**Methods:**

Competing risks methods were used to investigate the competing outcomes of death in neonatal care or discharge from the neonatal unit. The occurrence of one event prevents the other from occurring. This approach can be used to estimate the percentage of babies alive, or who have been discharged, over time.

**Results:**

A total of 20 571 very preterm babies were included. In the competing risks model, gestational age was adjusted for as a time-varying covariate, allowing the difference between weeks of gestational age to vary over time. The predicted percentage of death or discharge from the neonatal unit were estimated and presented graphically by week of gestational age. From these percentages, estimates of length of stay are provided as the number of days following birth and corrected gestational age at discharge.

**Conclusions:**

These results can be used in the counselling of parents about length of stay and the risk of mortality.

**What is already known on this topic?**

- Limited research has investigated length of stay in very preterm babies admitted for neonatal care.
- Parents are often told that their baby will be discharged home ‘around their due date’ but it is unclear whether reality reflects this estimate.

**What this study adds?**

- This study considers the risk of mortality and the length of stay of very preterm babies simultaneously, to present the full picture of neonatal care.
- For babies born at 24 and 25 weeks, length of stay should be considered alongside their risk of mortality.
- For babies born at 30 and 31 weeks, their median length of stay is a month less than the time remaining to their estimate date of delivery, indicating this anecdotal estimate of ‘home by their due date’ may be unhelpful in this group.

## Background

The ability to predict length of stay in neonatal care has become increasingly important as improvements in survival^1 2^ have led to more very preterm babies requiring long lengths of hospitalisation. Estimates of length of stay are necessary to facilitate conversations between parents and clinicians about a baby’s anticipated length of stay.

Previous research has often focused on investigating length of stay for babies who survive to discharge from neonatal care.^3–5^ Inclusion of babies who die while in neonatal care can make length of stay estimation complex.^6^ Other medical areas have recommended consideration of mortality and length of stay simultaneously as it can ‘reflect the reality or interrelation between the outcomes’.^7^ The exclusion of babies who die in neonatal care has been identified as a limitation of length-of-stay research in neonatal care.^8 9^

Currently estimates of length of stay for babies anticipated to survive are given as either ‘your baby will go home around the time they were due to be born’ or ‘when they are able to feed and keep themselves warm’. However, these statements are not evidence based and it is unclear if they are actually true. Irrespective of this, any results should be considered alongside the risk of mortality. Parents often report feeling anxious about whether they are ready to take their babies home, and information to support conversations about when this may happen may help alleviate some anxiety.^10^

Statistical methods recently introduced to neonatal research^11^ allow the simultaneous estimation of time to discharge or death. This paper aims to provide clinically useful estimates of length of stay and the risk of mortality to assist clinicians in consultation with parents.

## Methods

Data were obtained from the National Neonatal Research Database (NNRD), a population-based data source of information on admissions to neonatal care in England, created from information submitted by trusts to a commercial electronic patient record system.^12^

### Inclusion and exclusion criteria

Data were extracted on all singleton babies born at 24 to 31 weeks gestational age and admitted to neonatal units in England on the first day after delivery and discharged from 2011 to 2014. Babies born prior to 24 weeks gestational age were not included as their care is likely to relate to local policies, and there is a lack of consistency in approach to their management across the country.^13^

Babies were excluded if they were discharged home before 34 weeks postmenstrual age as it is not until this point that most babies acquire the ability to fully suck feed and maintain temperature stability.^14^ Babies that stayed in the neonatal unit longer than 6 months were also excluded. Exclusions were made for babies with unusual patterns of care including being discharged home having only received intensive care^15^ or being discharged having never received special care. These exclusions may be data errors or may represent a very different group of babies, including those receiving palliative care. Finally, babies were excluded if their final discharge was to another specialist service, for example, cardiac or surgical unit.

Daily data were available from the NNRD for babies throughout their time in neonatal care although babies could be transferred from neonatal care for other specialist care which does not provide data to the NNRD (eg, some surgical units) and then subsequently be transferred back into neonatal care. Days of care were imputed for these unobserved days.

Deaths in neonatal care and discharge home from neonatal care were considered as two competing events, that is, the occurrence of one event means the other cannot occur.

### Statistical analysis

A flexible parametric competing risks model^16–18^ was fitted in order to estimate the percentage of babies who were discharged or died in the neonatal unit over time.^19 20^ From this, estimates can be made of the percentage of deaths or discharges up to specific points in time. Completed weeks of gestational age at birth was included in the model as this is known to be important for both the prediction of mortality^21^ and length of stay.^6^ To allow for differences in the risk of mortality or discharge between the weeks of gestational age over time, time-dependent effects were included.^22^ Further methodological details for competing risks approaches, including their application in the estimation of neonatal length of stay, can be found elsewhere.^11 20^

The percentage of babies, by gestational age, dying or surviving to discharge from neonatal care was estimated over time and displayed graphically. Estimates of median length of stay can be derived from the point at which half of the events have occurred for babies who survived to discharge and for those who died in neonatal care.

## Results

There were 21 631 singleton babies born at 24–31 weeks gestational age discharged from neonatal care from 2011 to 2014. Babies were excluded if they were discharged home before 34 weeks postmenstrual age (n=205, 0.9%) or if they stayed in the neonatal unit longer than 6 months (n=199, 0.9%). Exclusions were made for unusual patterns of care defined as being discharged from neonatal care having only received intensive care (n=57, 0.3%) or discharged having never received special care (n=132, 0.6%). Babies were excluded if their final discharge was to another clinical location: another (specialist) hospital not reporting to the NNRD (n=293), surgical units (n=141), cardiac care (n=24) or an unknown location (n=9). A total of 20 571 (95%) babies remained in the analysis.

Summary characteristics of the included babies are provided in table 1. Over one million days of care were provided to this population of very preterm babies. Of the 20 571 babies in the analysis, 8.6% died during their time in neonatal care. Around 24% of babies were born at 31 weeks gestational age (table 1).

**Table 1:** Summary statistics of the singleton babies who were admitted for neonatal care at birth from 24 to 31 weeks from 2011 to 2014

Total babies admitted, n	
Gestational age, % (n)	
24	5.3 (1085)
25	6.0 (1244)
26	8.4 (1722)
27	9.8 (2006)
28	13.2 (2719)
29	14.8 (3041)
30	18.3 (3770)
31	24.2 (4984)
Sex of baby, % (n)	
Male	55.0 (11 308)
Female	45 (9243)
Indeterminate	<0.1 (20)
Total days of care, n	1 164 938
Birth weight (g), mean (SD)	1224 (371)
Died in neonatal care, % (n)	8.6 (1762)
24	39.1 (424)
25	22.4 (278)
26	16.1 (278)
27	11.9 (238)
28	7.5 (204)
29	3.9 (117)
30	2.9 (108)
31	2.3 (115)

### Gestational age analysis

**Figure 1:**
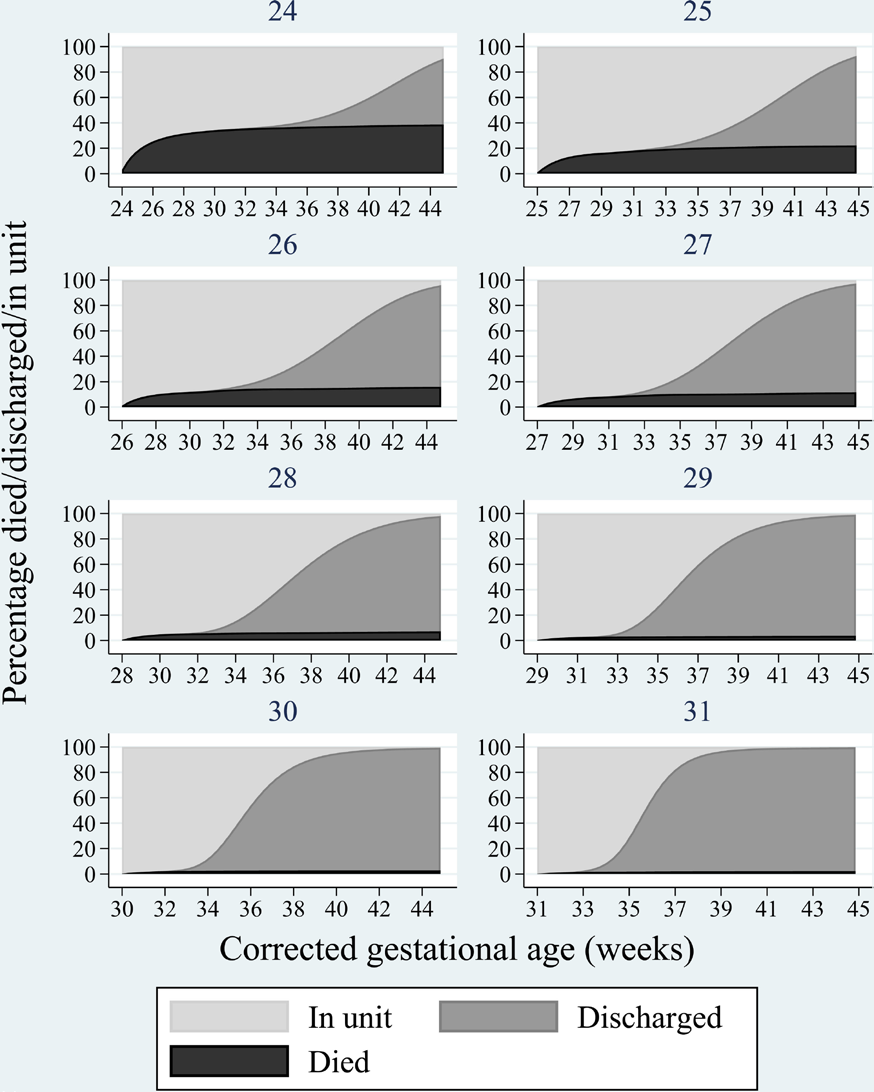
The percentage of babies that died before discharge (black); were discharged from neonatal care (grey) or remain in the neonatal unit (light grey).

The estimated percentages, from the flexible parametric competing risks model, are presented in graphical form as stacked plots (figure 1). The black area represents the percentage of babies who died in neonatal care, the dark grey area represents those discharged and the light grey area indicates the percentage who remain in the neonatal unit, over time. For example, for babies born at 24 weeks, the percentage of babies who had died by 30 days after birth (black area) was approximately 30% and no babies had been discharged (dark grey area). The rest of the babies remained in neonatal care (figure 1).

The median length of stay for babies was estimated by outcome of the baby and week of gestational age (table 2). The median length of stay is also presented as corrected gestational age at discharge. Babies born at 24 weeks who survived to discharge had a median length of stay of 123 days. This is slightly longer than the time remaining until their estimated date of delivery (discharge at 41.6 weeks corrected age). As week of gestational age increased the time to discharge decreased, and babies were discharged in advance of their due date. Babies born at 26–28 weeks had a median length of stay slightly shorter than the time remaining to their due date. However, babies born at 30 and 31 weeks were discharged home sooner, with a median length of stay around 30 days less than their due date.

**Table 2:** Median length of stay and median corrected age at discharge with range (25th, 75th centile) by outcome

Gestational age	Days to due date	Length of stay (days) of discharges	Corrected gestational age at discharge	Length of stay (days) of deaths
24	112	123 (104, 139)	41.6 (38.9, 43.9)	9 (3, 21)
25	105	107 (88, 125)	40.3 (37.6, 42.9)	10 (3, 27)
26	98	92 (74, 109)	39.1 (36.6, 41.6)	10 (3, 30)
27	91	79 (63, 96)	38.3 (36, 40.7)	11 (4, 35)
28	84	66 (52, 82)	37.4 (35.4, 39.7)	10 (3, 18)
29	77	53 (43, 66)	36.6 (35.1, 38.4)	8 (2, 40)
30	70	42 (34, 52)	36.0 (34.9, 37.4)	4 (4, 17)
31	63	34 (28, 41)	35.9 (35, 36.9)	7 (1, 7)

Babies dying while in neonatal care had a median length of stay of around ≤10 days, indicating that half of deaths occur in the first 10 days after birth.

## Discussion

This research has provided estimates of median length of stay while also considering mortality for singleton babies born very preterm. These estimates can be used in clinical practice to aid the counselling of parents about length of stay. For example, for a baby born at 26 weeks gestational age around half of deaths have occurred in the first 10 days (table 2). At around 10 days of life, and using their clinical judgement, a clinician could explain to a parent that the risk of mortality has reduced, but that their baby could be in hospital for a long time. The estimate of median length of stay for a baby of these characteristics is 92 days (82 days by day 10) but we would suggest that clinicians use a more general description, for example, ‘around two and a half months’ or in terms of their due date: ‘around a week before their due date’, to reflect that there is uncertainty in this estimate. Future qualitative research should focus on the issues of how to communicate the risk of mortality and length of stay to parents.

Anecdotally, parents are often told their baby will go home ‘around their due date’ and this research demonstrates that this may not be the case. Babies born at 24 and 25 weeks of gestational age who survive to discharge have the longest median length of stay, staying around 123 and 107 days, respectively. For these babies, saying they may be discharged ‘around their due date’ is close to their median length of stay. However, for babies born at 30 and 31 weeks gestational age, their median length of stay is around a month shorter than the time remaining to their estimated due date. Therefore, this phrase should be used with caution as it seems that this may not accurately reflect length of stay for many very preterm babies.

Parents have reported that information about likely discharge dates improved their understanding of their baby’s progress and prepared them for discharge.^23^ However, this information should be given at an appropriate time, in an appropriate way and supplemented with clinical judgement. Around half of the deaths occur in the first 10 days of life, and clinicians should consider this when counselling around length of stay. The estimates provided in this work are intended to complement and facilitate clinician knowledge, rather than replace it.

### Strengths and limitations

This analysis was adjusted for gestational age alone. While other factors may be important for the estimation of length of stay,^6^ it is helpful if statistical models are simple, informative and easy to use within a clinical setting. In attempts to predict neonatal mortality, risk scores have been created which have subsequently needed to be simplified because they were too ‘cumbersome to use’ in practice.^24 25^

This study is one of the largest studies to investigate the prediction of length of stay in neonatal care. A strength of this work is that these results have been produced on a national basis, without biases arising from differences between networks of hospitals or individual neonatal units due to local discharge practices within units or networks. All neonatal units in England contributed their data to this study allowing consideration of the total care received by each baby, even across multiple units and transfers, without loss to follow-up. However, as the results are population based we did not consider that units may have individual approaches to length of stay and discharge planning. We did not investigate individual units as small numbers of babies, particularly at the earliest weeks of gestational age, at specific units would make estimation of their length of stay imprecise. For the same reasons we were unable to investigate specific subgroups of babies, such as those who require surgery, but future work should consider this area.

Babies discharged to receive care in other services were excluded from this work. These babies will potentially have a length of stay longer than that seen in the data reported to the NNRD. However, these babies represented a small number of discharges from neonatal care (n=467).

There has been limited work investigating neonatal length of stay in the UK, but another small study investigating length of stay in four neonatal units in the Southwest of England found similar results to this work (the ‘Train-to-Home’ package), with babies born from 27 to 33 weeks being discharged 3–4 weeks in advance of their estimated date of delivery.^26^ Estimates of length of stay from The Neonatal Survey from 2005 to 2007, a study of neonatal intensive care in the East Midlands and Yorkshire, also found similar results to those presented in this work.^5^ This allows the potential for clinicians to offer more accurate information to parents than just telling them that their baby will go home ‘around their due date’.

### Future work

Estimates of total length of stay can be useful for parental counselling, and they are also helpful in clinician discussions about a baby. However, they do not provide the entire picture of neonatal care. While in neonatal care a baby will need varying levels of care^15^ and this can be incorporated into length-of-stay estimates. Estimates incorporating information about levels of care may be more informative for service planning and the commissioning of care. We are investigating this in further detail and initial results have been published elsewhere.^27^ Future work should also investigate differences in length of stay between different regions and different subgroups of babies, for example, babies discharged home on oxygen.

Singleton babies born very preterm have been investigated in this work as it is unlikely to be possible to predict length of stay for singleton and multiple babies simultaneously.^9^ The singleton, very preterm population is somewhat homogenous in terms of their prematurity which is likely to be the most important determining factor of their length of stay.^6^ Babies born after 32 weeks gestational age may need an analysis stratified by their clinical condition, although this may still be problematic as even babies with similar clinical conditions have been seen to have varying lengths of stay within a single unit.^28^

There is no evidence to suggest on the optimum length of stay in a neonatal unit before discharge, nor evidence that a short length of stay should be a desirable aim.^9^ Following an early discharge home, babies may require admission to paediatric care within a short period of time, whereas keeping them in the neonatal unit a little longer may have minimised this risk. Future research should link neonatal care with other outcomes, including subsequent admission to paediatric care, to investigate the benefits and harms of early versus late discharge from neonatal care.

## Conclusion

The estimation of length of stay in neonatal care should also consider the risk of mortality, especially for the very preterm. In this work, appropriate statistical methods have been used to provide estimates of length of stay which can be used by clinicians to aid the timing, and content, of discussions with parents.

## References

[1] Field DJ, Dorling JS, Manktelow BN, et al Survival of extremely premature babies in a geographically defined population: prospective cohort study of 1994-9 compared with 2000-5. BMJ 2008;336:1221–3. 10.1136/bmj.39555.670718.BE 18469017 PMC2405852

[2] Manktelow BN, Seaton SE, Field DJ, et al Population-based estimates of in-unit survival for very preterm infants. Pediatrics 2013;131:e425–32. 10.1542/peds.2012-2189 23319523

[3] Altman M, Vanpée M, Cnattingius S, et al Moderately preterm infants and determinants of length of hospital stay. Arch Dis Child Fetal Neonatal Ed 2009;94:F414–8. 10.1136/adc.2008.153668 19465411

[4] Lee HC, Bennett MV, Schulman J, et al Accounting for variation in length of NICU stay for extremely low birth weight infants. J Perinatol 2013;33:872–6. 10.1038/jp.2013.92 23949836 PMC3815522

[5] Manktelow B, Draper ES, Field C, et al Estimates of length of neonatal stay for very premature babies in the UK. Arch Dis Child Fetal Neonatal Ed 2010;95:F288–92. 10.1136/adc.2009.168633 20530099

[6] Seaton SE, Barker L, Jenkins D, et al What factors predict length of stay in a neonatal unit: a systematic review. BMJ Open 2016;6(10):e010466 10.1136/bmjopen-2015-010466 PMC5073598 27797978

[7] Taylor SL, Sen S, Greenhalgh DG, et al A competing risk analysis for hospital length of stay in patients with burns. JAMA Surg 2015;150:450–6. 10.1001/jamasurg.2014.3490 25761045 PMC4968081

[8] Bender GJ, Koestler D, Ombao H, et al Neonatal intensive care unit: predictive models for length of stay. J Perinatol 2013;33:147–53. 10.1038/jp.2012.62 22678140 PMC4073289

[9] Lee HC, Bennett MV, Schulman J, et al Estimating Length of Stay by Patient Type in the Neonatal Intensive Care Unit. Am J Perinatol 2016;33:751–7. 10.1055/s-0036-1572433 26890437

[10] Turner M, Winefield H, Chur-Hansen A The emotional experiences and supports for parents with babies in a neonatal nursery. Adv Neonatal Care 2013;13:438–46. 10.1097/ANC.0000000000000030 24300964

[11] Hinchliffe SR, Seaton SE, Lambert PC, et al Modelling time to death or discharge in neonatal care: an application of competing risks. Paediatr Perinat Epidemiol 2013;27:426–33. 10.1111/ppe.12053 23772944

[12] Gale C, Morris I; Neonatal Data Analysis Unit (NDAU) Steering Board. The UK National Neonatal Research Database: using neonatal data for research, quality improvement and more. Arch Dis Child Educ Pract Ed 2016;101:216–8. 10.1136/archdischild-2015-309928 26968617 PMC4975807

[13] Smith L, Draper ES, Manktelow BN, et al Comparing regional infant death rates: the influence of preterm births <24 weeks of gestation. Arch Dis Child Fetal Neonatal Ed 2013;98:F103–7. 10.1136/fetalneonatal-2011-301359 22684158 PMC3582045

[14] Phibbs CS, Schmitt SK Estimates of the cost and length of stay changes that can be attributed to one-week increases in gestational age for premature infants. Early Hum Dev 2006;82:85–95. 10.1016/j.earlhumdev.2006.01.001 16459031 PMC1752207

[15] British Association of Perinatal Medicine. BAPM categories of care 2011 http://www.bapm.org/publications/ (Last accessed: 20/02/2017).

[16] Royston P A strategy for modelling the effect of a continuous covariate in medicine and epidemiology. Stat Med 2000;19:1831–47. 10867674 10.1002/1097-0258(20000730)19:14<1831::aid-sim502>3.0.co;2-1

[17] Royston P, Ambler G, Sauerbrei W The use of fractional polynomials to model continuous risk variables in epidemiology. Int J Epidemiol 1999;28:964–74. 10597998 10.1093/ije/28.5.964

[18] Durrleman S, Simon R Flexible regression models with cubic splines. Stat Med 1989;8:551–61. 2657958 10.1002/sim.4780080504

[19] Royston P, Parmar MK Flexible parametric proportional-hazards and proportional-odds models for censored survival data, with application to prognostic modelling and estimation of treatment effects. Stat Med 2002;21:2175–97. 10.1002/sim.1203 12210632

[20] Hinchliffe SR, Lambert PC Flexible parametric modelling of cause-specific hazards to estimate cumulative incidence functions. BMC Med Res Methodol 2013;13:1–14. 10.1186/1471-2288-13-13 23384310 PMC3614517

[21] Medlock S, Ravelli AC, Tamminga P, et al Prediction of mortality in very premature infants: a systematic review of prediction models. PLoS One 2011;6:e23441 10.1371/journal.pone.0023441 21931598 PMC3169543

[22] Lambert PC, Royston P Further development of flexible parametric models for survival analysis. Stata Journal 2009;9:265–90.

[23] Ingram JC, Powell JE, Blair PS, et al Does family-centred neonatal discharge planning reduce healthcare usage? A before and after study in South West England. BMJ Open 2016;6:e010752 10.1136/bmjopen-2015-010752 PMC4800152 26966062

[24] Richardson DK, Gray JE, McCormick MC, et al Score for Neonatal Acute Physiology: a physiologic severity index for neonatal intensive care. Pediatrics 1993;91:617–23. 8441569

[25] Richardson DK, Corcoran JD, Escobar GJ, et al SNAP-II and SNAPPE-II: Simplified newborn illness severity and mortality risk scores. J Pediatr 2001;138:92–100. 11148519 10.1067/mpd.2001.109608

[26] Fleming PJ, Ingram J, Johnson D, et al Estimating discharge dates using routinely collected data: improving the preparedness of parents of preterm infants for discharge home. Arch Dis Child Fetal Neonatal Ed 2017;102 10.1136/archdischild-2016-310944 PMC5339560 27698193

[27] Seaton SE, Barker L, Draper ES, et al Modelling Neonatal Care Pathways for Babies Born Preterm: An Application of Multistate Modelling. PLoS One 2016;11:e0165202 10.1371/journal.pone.0165202 27764232 PMC5072657

[28] Shetty S, Kennea N, Desai P, et al Length of stay and cost analysis of neonates undergoing surgery at a tertiary neonatal unit in England. Ann R Coll Surg Engl 2016;98:56–60. 10.1308/rcsann.2016.0034 26688402 PMC5234397

